# Prior statin and short‐term outcomes of primary intracerebral hemorrhage: From a large‐scale nationwide longitudinal registry

**DOI:** 10.1111/cns.13868

**Published:** 2022-05-23

**Authors:** Guangshuo Li, Shang Wang, Yunyun Xiong, Hongqiu Gu, Kaixuan Yang, Xin Yang, Chunjuan Wang, Chuanying Wang, Zixiao Li, Xingquan Zhao

**Affiliations:** ^1^ Vascular Neurology, Department of Neurology, Beijing Tiantan Hospital Capital Medical University Beijing China; ^2^ Neurocardiology Center, Department of Neurology, Beijing Tiantan Hospital Capital Medical University Beijing China; ^3^ Chinese Institute of Brain Research Beijing China; ^4^ China National Clinical Research Center for Neurological Diseases Beijing China; ^5^ National Center for Healthcare Quality Management in Neurological Diseases Beijing China; ^6^ Beijing Key Laboratory of Translational Medicine for Cerebrovascular Disease Beijing China; ^7^ Center for Stroke, Beijing Institute for Brain Disorders Beijing China

**Keywords:** cerebral hemorrhage, hydroxymethylglutaryl‐CoA reductase inhibitors, mortality, treatment outcome

## Abstract

**Introduction:**

The relationship between statins and intracerebral hemorrhage outcomes is unclear.

**Aim:**

We aimed to compare the in‐hospital mortality and evacuation of intracranial hematoma rates in patients with primary intracerebral hemorrhage between prior statin users and nonusers.

**Results:**

The final study population included 66,263 patients. Multivariable logistics analyses showed that prior statin use was not associated with in‐hospital mortality for primary intracerebral hemorrhage (adjusted odd ratio 0.78, 95% CI 0.61–1.01), but reduced the proportion of patients undergoing evacuation of intracranial hematoma (adjusted odd ratio 0.70, 95% CI 0.61–0.82). Propensity score matching analyses yielded similar results.

**Conclusion:**

Prior statin use was not associated with in‐hospital mortality but did reduce evacuation of intracranial hematoma rates.

## Graphical abstract

The relationship between statins and intracerebral hemorrhage outcomes is unclear. We aimed to compare the in‐hospital mortality and the rates of evacuation of intracranial hematoma in patients with primary intracerebral hemorrhage between prior statins users and non‐users. A retrospective study was conducted and data were derived from the Chinese Stroke Center Consortium (CSCA). Patients aged 50 years or older with a diagnosis of primary intracerebral hemorrhage were included. Demographic data, medical history, NIHSS, and Glasgow coma scale (GCS) score were collected. Outcome measures included death at discharge and evacuation of intracranial hematoma. Multivariable logistic regression analyses and propensity score matching analyses were used to investigate the relationship between prior statins use and the clinical outcomes. The final study population included 66,263 patients. Multivariable logistics analyses showed that prior statins use was not associated with in‐hospital mortality for primary intracerebral hemorrhage (adjusted OR 0.78, 95% CI 0.61–1.01), but reduced the proportion of patients undergoing evacuation of intracranial hematoma (adjusted OR 0.70, 95% CI 0.61–0.82), and propensity score matching analyses yielded similar results. Prior statins use is not associated with in‐hospital mortality, but reduced the rates of evacuation of intracranial hematoma.

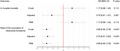

## 1 INTRODUCTION

Primary intracerebral hemorrhage (ICH) has a higher mortality and disability rate than other types of stroke, with an independence rate ranging from 12% to 39%.^1^, ^2^, ^3^ Although statins can lower serum lipid levels and stabilize atherosclerotic plaques to reduce the risk of ischemic stroke recurrence, their effects on ICH remain unclear.

The Stroke Prevention by Aggressive Reduction in Cholesterol Levels (SPARCL) trial enrolled 4731 patients and showed that 80 mg atorvastatin doubled the risk of ICH.^4^ A nationwide case–control study that included 7696 cases of ICH and 14,670 controls reported that statins were associated with a 32% lower risk of incident ICH.^5^ Cohort studies have revealed that statins could reduce by threefold the odds of mortality in patients diagnosed with ICH.^6^ A large‐scale study (*n* = 17,980) based on the National Health Insurance Research Database in Taiwan also showed that patients prescribed with statins after ICH had a lower risk of all‐cause mortality and cardiovascular death.^7^ Currently, there is no consensus regarding the effect of statin use prior to ICH.^8^ The ongoing Statins in Intracerebral Hemorrhage (SATURN) trial (ClinicalTrials.gov Identifier: NCT03936361), specifically designed to investigate the efficacy and safety of continuation vs. discontinuation of statin drugs after primary lobar ICH, will provide more information on this topic.

In the current study, we compared the in‐hospital mortality and evacuation rates of intracranial hematoma between statin users and non‐statin users to investigate the relationship between prior statin treatment and ICH clinical outcomes. Moreover, we conducted an additional analysis to investigate the relationship between prior statin treatment and ICH outcomes in subgroups of patients with a history of stroke, as well as those with and without a history of dyslipidemia.

## 2 SUBJECTS AND METHODS

### 2.1 Participants

We derived data from the China Stroke Center Alliance (CSCA), a national multicenter registry cohort initiated to promote the quality of the diagnosis and treatment of stroke in 1476 hospitals in China.^9^ From 2015 to 2019, 1,006,798 patients with stroke or transient ischemic attack were consecutively enrolled in the CSCA,^10^ of which 85,705 were diagnosed with ICH. In the current study, the inclusion criteria were a) age of >50 years and b) a diagnosis of ICH. Patients with incomplete clinical, imaging, or laboratory information, particularly statin medication histories, were excluded.

Data were collected from each center. All identifiers for each patient were removed before statistical analysis, and the data were only accessible under the surveillance of the China National Clinical Research Center for Neurological Diseases to protect the privacy and confidentiality of the enrolled patients. The participating hospitals received research approval to collect data for the CSCA study without requiring individual patient informed consent under the common rule, or a waiver of authorization and exemption from subsequent review by their institutional review board.

### 2.2 Clinical characteristics and outcome assessment

A Web‐based patient data collection and management tool (Medicine Innovation Research Center, Beijing, China) was used to collect the demographic and clinical characteristics of the participants. Based on the medication history before admission (oral administration of statin drugs at the time of the index ICH), we categorized the enrolled patients into two groups (1): Patients with prior statin therapy before ICH and (2) patients without prior statin therapy before ICH. Statin therapy refers to oral statin drugs such as lovastatin, simvastatin, pravastatin, fluvastatin, atorvastatin, rosuvastatin, and pivastatin. The discharge destination was recorded for subsequent analyses. The primary outcome was in‐hospital mortality based on the discharge destination, while the secondary outcome was the rate of intracranial hematoma evacuation.

### 2.3 Subgroup analyses

Subgroup analyses were conducted in patients with a history of (1) stroke, (2) ischemic stroke, (3) hemorrhagic stroke, or (4) dyslipidemia, as well as in (5) patients without a history of dyslipidemia, to determine the association of prior statin use with in‐hospital mortality and evacuation rates of intracranial hematoma.

### 2.4 Statistical analysis

Data were tested for normal distribution using the Kolmogorov–Smirnov test. In the univariate analyses, comparison between prior statin users and non‐users was performed in normally distributed data using the Student's *t*‐test and in skewed data using the Mann–Whitney U test. Categorical variables were analyzed using the Pearson's χ^2^ or Fisher's exact test, as appropriate. Considering the extensive sample size, using an absolute standardized difference of ≥10 was more appropriate than *p* < 0.05 in demonstrating a clinically significant difference in baseline characteristics. Propensity score matching (PSM)^11^ was used to balance the baseline characteristics of statin users and non‐users. For the PSM analysis, a 1:1 matching was performed based on the nearest‐neighbor matching algorithm with a caliper width of 0.25 of the propensity score using the medical history of hypertension, diabetes mellitus, atrial fibrillation, myocardial infarction, heart failure, ischemic stroke, cerebral hemorrhage, medication history of antiplatelet agents, anticoagulant agents, antihypertensive agents, antidiabetic agents, fasting plasma glucose, systolic blood pressure, diastolic blood pressure, and international normalized ratio as covariates. Multiple logistic regression analyses were used to test the correlation of statin use with the in‐hospital mortality and evacuation rates of intracranial hematoma. Statistical significance was defined as a two‐sided *p* < 0.05. All analyses were performed using the SAS software (version 9.4; SAS Institute, Inc.).

## 3 RESULTS

### 3.1 Patient characteristics

A total of 66,263 patients were included in the study. Among the enrolled patients, the mean age was 66.4 years (SD 10.3), and 40,360 (60.9%) were men. In total, 4190 (6.3%) patients were included in the prior statin group and 62,073 patients were included in the non‐user group. Table 1 shows a comparison of baseline characteristics and clinical outcomes between statin users and non‐users. Compared with non‐users, statin users were more likely to have medical comorbidities, including hypertension (86.3% vs. 71.8%), diabetes mellitus (24.0% vs. 9.2%), dyslipidemia (25.3% vs. 2.6%), atrial fibrillation (5.1% vs. 1.4%), myocardial infarction (4.1% vs 0.7%), heart failure (1.5% vs. 0.4%), ischemic stroke (43.9% vs. 11.9%), and cerebral hemorrhage (31.6% vs. 17.1%), as well as a medication history, including antiplatelet agents (54.5% vs. 4.4%), anticoagulant agents (11.9% vs. 1.3%), antihypertensive agents (83.8% vs 47.3%), and antidiabetic agents (22.5% vs. 6.6%).

**TABLE 1:** Demographic and clinical characteristics between prior statins users and non‐users before PSM

Variables	Total(*N* = 66,263)	Prior statins users(*N* = 4,190)	Non‐users(*N* = 62,073)	ASD value
Demographic				
Age (Mean ± SD, years)	66.4 ± 10.3	67.3 ± 10.1	66.3 ± 10.3	9.8
Male (*n*, %)	40,360 (60.9)	2613 (62.4)	37,747 (60.8)	3.3
NIHSS score at admission (Mean ± SD)	8.7 ± 9.0	8.8 ± 9.1	8.7 ± 9.0	1.1
GCS at admission (Mean ± SD)	11.4 ± 4.1	11.1 ± 4.3	11.4 ± 4.1	7.1
Medical history				
Hypertension (*n*, %)	48,192 (72.7)	3616 (86.3)	44,576 (71.8)	36.2
Diabetes mellitus (*n*, %)	6697 (10.1)	1007 (24.0)	5690 (9.2)	40.6
Dyslipidemia (*n*, %)	2704 (4.1)	1061 (25.3)	1643 (2.6)	69.3
Atrial fibrillation (*n*, %)	1097 (1.7)	214 (5.1)	883 (1.4)	21.0
Myocardial infarction (*n*, %)	634 (1.0)	171 (4.1)	463 (0.7)	22.4
Heart failure (*n*, %)	313 (0.5)	64 (1.5)	249 (0.4)	11.4
Ischemic stroke (*n*, %)	9213 (13.9)	1838 (43.9)	7375 (11.9)	76.4
Cerebral hemorrhage (*n*, %)	11,957 (18.0)	1322 (31.6)	10,635 (17.1)	34.3
Smoking (*n*, %)	21,260 (32.1)	1476 (35.2)	19,784 (31.9)	7.0
Alcoholism (*n*, %)	15,456 (23.3)	1030 (24.6)	14,426 (23.2)	3.3
Medication history				
Antiplatelet agents (*n*, %)	5014 (7.6)	2284 (54.5)	2730 (4.4)	131.6
Anticoagulant agents (*n*, %)	1298 (2.0)	499 (11.9)	799 (1.3)	43.7
Antihypertensive agents (*n*, %)	32,860 (49.6)	3513 (83.8)	29,347 (47.3)	83.2
Antidiabetic agents (*n*, %)	5028 (7.6)	943 (22.5)	4085 (6.6)	46.3
Laboratory findings				
LDL‐C (Mean ± SD, mmol/L)	2.8 ± 1.5	2.8 ± 1.8	2.8 ± 1.5	<0.1
Total cholesterol (Mean ± SD, mmol/L)	1.0 ± 1.9	1.0 ± 1.9	1.0 ± 1.9	<0.1
Triglyceride (Mean ± SD, mmol/L)	0.4 ± 1.1	0.5 ± 1.3	0.4 ± 1.1	8.3
Fasting plasma glucose (Mean ± SD, mmol/L)	6.6 ± 2.8	6.9 ± 3.2	6.5 ± 2.8	13.3
Systolic blood pressure (Mean ± SD, mmHg)	164.8 ± 27.6	160.4 ± 26.3	165.1 ± 27.7	17.4
Diastolic blood pressure (Mean ± SD, mmHg)	94.4 ± 16.0	92.2 ± 15.5	94.5 ± 16.1	14.6
INR (Mean ± SD)	1.2 ± 1.1	1.4 ± 1.2	1.2 ± 1.1	17.4
In‐hospital outcome				
In‐hospital mortality (*n*, %)	1534 (2.3)	112 (2.7)	1422 (2.3)	2.6
Evacuation of intracranial hematoma (*n*, %)	6698 (10.1)	302 (7.2)	6396 (10.3)	11.0

Abbreviations: ASD, absolute standard deviation; GCS, glasgow coma score; INR, international normalized ratio; LDL‐C, low‐density lipoprotein cholesterol; NIHSS, national institutes of health stroke scale; PSM, propensity score match.

### 3.2 Prior statin use and outcomes

The in‐hospital mortality rates were similar between prior statin users and non‐users (2.7% vs 2.3%). The evacuation rate of intracranial hematoma was also significantly reduced in prior statin users compared to non‐users (7.2% vs. 10.3%).

**FIGURE 1:**
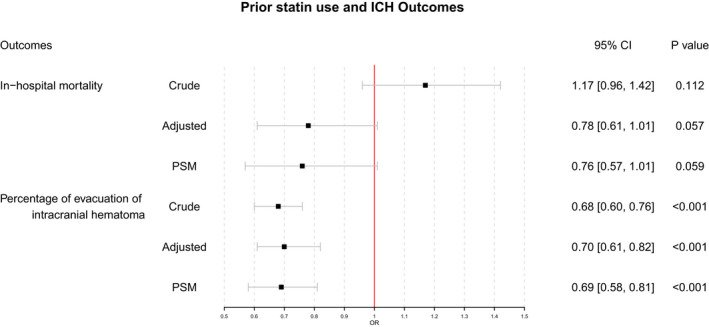
Logistics regression analyses of clinical outcomes. Adjusted: Adjusted for medical history of hypertension, diabetes mellitus, dyslipidemia, atrial fibrillation, myocardial infarction, heart failure, ischemic stroke, cerebral hemorrhage, mediation history of antiplatelet agents, anticoagulant agents, antihypertensive agents, antidiabetic agents, fasting plasma glucose, systolic blood pressure, diastolic blood pressure and INR. PSM, propensity score matching

Logistic regression analyses showed that prior statin use was not associated with in‐hospital mortality in either the unadjusted or adjusted model (crude OR 1.17, 95% CI 0.96–1.42, *p* = 0.11; adjusted OR 0.78, 95% CI 0.61–1.01, *p* = 0.06). Prior statin use was associated with a lower evacuation rate of intracranial hematoma, both in the unadjusted and adjusted models (crude OR 0.68, 95% CI 0.60–0.76), *p* < 0.001; adjusted OR 0.70, 95% CI 0.61–0.82, *p* < 0.001) (Figure 1).

### 3.3 Subgroup analyses

**FIGURE 2:**
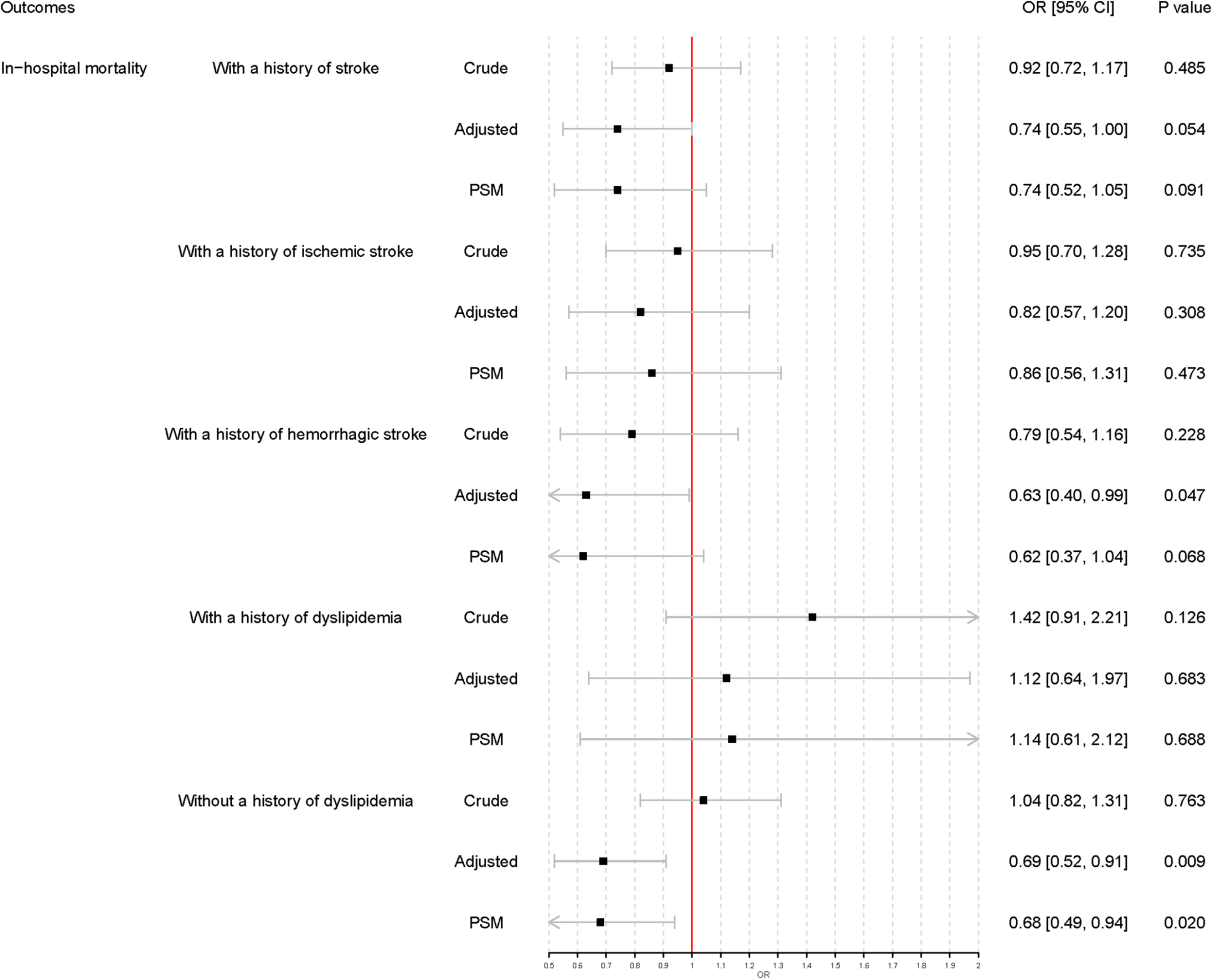
Subgroup analysis of in‐hospital mortality. Adjusted: Adjusted for medical history of hypertension, diabetes mellitus, dyslipidemia, atrial fibrillation, myocardial infarction, heart failure, ischemic stroke, cerebral hemorrhage, mediation history of antiplatelet agents, anticoagulant agents, antihypertensive agents, antidiabetic agents, fasting plasma glucose, systolic blood pressure, diastolic blood pressure, and INR. PSM, propensity score matching

**FIGURE 3:**
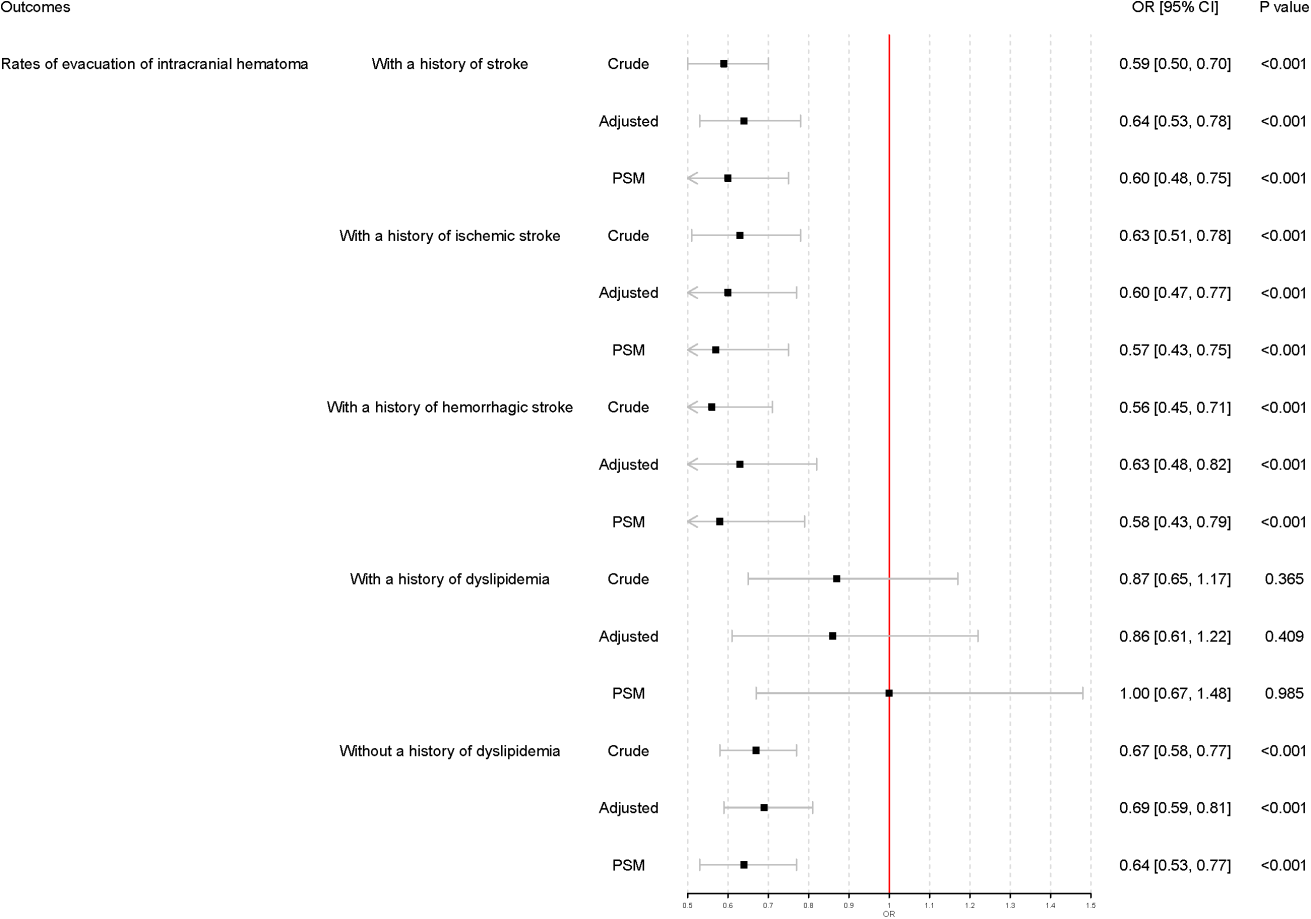
Subgroup analysis of evacuation of intracranial hematoma. Adjusted: Adjusted for medical history of hypertension, diabetes mellitus, dyslipidemia, atrial fibrillation, myocardial infarction, heart failure, ischemic stroke, cerebral hemorrhage, mediation history of antiplatelet agents, anticoagulant agents, antihypertensive agents, antidiabetic agents, fasting plasma glucose, systolic blood pressure, diastolic blood pressure and INR. PSM, propensity score matching

Subgroup analyses were conducted in patients with a history of stroke, ischemic stroke, and hemorrhagic stroke. Prior statin use was not associated with in‐hospital mortality in the three subgroup analyses. Prior statin use was also associated with lower evacuation rates of intracranial hematoma among patients with a history of stroke (adjusted OR 0.64, 95% CI 0.53–0.78), ischemic stroke (adjusted OR 0.60, 95% CI 0.47–0.77), and hemorrhagic stroke (adjusted OR 0.63, 95% CI 0.48–0.82) (Figures 2 and 3).

Additional subgroup analyses were conducted in patients with or without a history of dyslipidemia. Prior statin use failed to demonstrate an association with either in‐hospital mortality or evacuation rates of intracranial hematoma in patients with a history of dyslipidemia. In patients without dyslipidemia, subgroup analyses showed that prior statin use was associated with in‐hospital mortality (adjusted OR 0.69, 95% CI 0.52–0.91) and evacuation rates of intracranial hematoma (adjusted OR 0.69, 95% CI 0.59–0.81) (Figures 2 and 3).

### 3.4 Results after PSM

All covariates were well‐balanced through the PSM (Table 2). Additional logistic regression analyses were conducted based on the results of PSM and demonstrated that prior statin use was not associated with in‐hospital mortality (*p* = 0.059) but was associated with a lower rate of intracranial hematoma evacuation (OR 0.69, 95% CI 0.58–0.81, *p* < 0.001) (Figure 1). Additional PSM revealed subgroup analysis results that were similar to the prior logistic regression analysis results (Figures 2 and 3).

**TABLE 2:** Demographic and clinical characteristics between prior statins users and non‐users after PSM

Variables	Total(*N* = 7038)	Prior statins users(*N* = 3519)	Non‐users(*N* = 3519)	ASD value
Demographic				
Age (Mean ± SD, years)	67.2 ± 10.0	67.2 ± 10.0	67.3 ± 9.9	1.0
Male (*n*, %)	4352 (61.8)	2166 (61.6)	2186 (62.1)	1.0
NIHSS score at admission (Mean ± SD)	8.9 ± 9.2	8.7 ± 9.0	9.1 ± 9.4	4.3
GCS at admission (Mean ± SD)	11.1 ± 4.2	11.1 ± 4.3	11.2 ± 4.2	2.4
Medical history				
Hypertension (*n*, %)	6048 (85.9)	3018 (85.8)	3030 (86.1)	0.9
Diabetes mellitus (*n*, %)	1497 (21.3)	772 (21.9)	725 (20.6)	3.2
Dyslipidemia (*n*, %)	1501 (21.3)	759 (21.6)	742 (21.1)	1.2
Atrial fibrillation (*n*, %)	340 (4.8)	171 (4.9)	169 (4.8)	0.5
Myocardial infarction (*n*, %)	245 (3.5)	126 (3.6)	119 (3.4)	1.1
Heart failure (*n*, %)	69 (1.0)	36 (1.0)	33 (0.9)	1.0
Ischemic stroke (*n*, %)	2977 (42.3)	1483 (42.1)	1494 (42.5)	0.8
Cerebral hemorrhage (*n*, %)	2265 (32.2)	1122 (31.9)	1143 (32.5)	1.3
Smoking (*n*, %)	2421 (34.4)	1230 (35.0)	1191 (33.8)	2.5
Alcoholism (*n*, %)	1648 (23.4)	850 (24.2)	798 (22.7)	3.5
Medication history				
Antiplatelet agents (*n*, %)	3560 (50.6)	1800 (51.2)	1760 (50.0)	2.4
Anticoagulant agents (*n*, %)	744 (10.6)	398 (11.3)	346 (9.8)	4.9
Antihypertensive agents (*n*, %)	5891 (83.7)	2931 (83.3)	2960 (84.1)	2.2
Antidiabetic agents (*n*, %)	1403 (19.9)	727 (20.7)	676 (19.2)	3.8
Laboratory findings				
LDL‐C (Mean ± SD, mmol/L)	2.8 ± 1.8	2.8 ± 1.9	2.9 ± 1.7	5.5
Total cholesterol (Mean ± SD, mmol/L)	1.0 ± 1.9	1.0 ± 1.9	0.9 ± 1.9	5.3
Triglyceride (Mean ± SD, mmol/L)	0.5 ± 1.4	0.5 ± 1.3	0.5 ± 1.6	<0.1
Fasting plasma glucose (Mean ± SD, mmol/L)	6.9 ± 3.1	6.9 ± 3.2	6.9 ± 3.0	<0.1
Systolic blood pressure (Mean ± SD, mmHg)	160.4 ± 26.5	160.3 ± 26.5	160.4 ± 26.6	0.4
Diastolic blood pressure (Mean ± SD, mmHg)	92.2 ± 15.6	92.2 ± 15.5	92.2 ± 15.7	<0.1
INR (Mean ± SD)	1.4 ± 1.3	1.4 ± 1.2	1.4 ± 1.3	<0.1
In‐hospital outcome				
In‐hospital mortality (*n*, %)	194 (2.8)	84 (2.4)	110 (3.1)	4.3
Evacuation of intracranial hematoma (*n*, %)	614 (8.7)	255 (7.2)	359 (10.2)	10.7

Abbreviations: ASD, absolute standard deviation; GCS, glasgow coma score; INR, international normalized ratio; LDL‐C, low‐density lipoprotein cholesterol; NIHSS, national institutes of health stroke scale; PSM, propensity score match.

## 4 DISCUSSION

Our study found that prior statin use was not associated with in‐hospital mortality but was associated with a significantly reduced risk of intracranial hematoma evacuation in patients with ICH. Subgroup analyses showed that prior statin use was associated with lower evacuation rates of intracranial hematoma in patients with a history of ischemic stroke or hemorrhagic stroke, as well as in patients without a history of dyslipidemia. Currently, our study employed the largest sample size to investigate the relationship between statin use and clinical outcomes in patients with ICH. CSCA is a national accumulated electronic medical record database that includes 1,006,798 patients from 1476 hospitals in China and is representative of the characteristics of cerebrovascular disease. A sufficient sample size and systematic statistical analysis methods enhanced the robustness of the results of the current study.

Our study failed to show a correlation between prior statin use and in‐hospital mortality in ICH patients, which is consistent with the results of a meta‐analysis of 12 studies including 6961 patients.^12^ Moreover, a meta‐analysis including 26,757 patients from 12 studies demonstrated that prior statin use before ICH was not associated with mortality.^13^ A post‐hoc analysis of SPARCL showed that statin use had no impact on the severity, functional independence, or mortality of ICH.^14^

Our study showed significantly reduced evacuation rates of intracranial hematoma in patients with prior statin use. Neurosurgeons would remove the hematoma by surgery in patients with a severe clinical status, including large hematoma volume, hematoma expansion, neurological deterioration, or cerebral hernia. Hence, evacuation of intracranial hematoma could represent severe cases of ICH, including large hematoma, hematoma expansion, and perihematomal edema (PHE) or cerebral hernia.^15^ Therefore, lower rates of evacuation of intracranial hematoma indicate a milder severity of ICH in statin users. This finding is consistent with that of a previous study enrolling 2,466 patients with ICH based on the Registry of the Canadian Stroke Network.^16^ A post‐hoc analysis of 73 ICH patients enrolled in the Intracerebral Hemorrhage Acutely Decreasing Arterial Pressure Trial (ICH ADAPT) showed similar median hematoma volumes in statin users compared to non‐treated patients.^17^ A multicenter study also demonstrated that prior statin use had no effect on hematoma growth.^18^ PHE volumes were lower in prior statin users than in non‐users.^12^, ^15^, ^19^ Hence, it is hypothesized that lower rates of evacuation of intracranial hematoma in prior statin users may be due to less PHE volume.

Perihematomal edema is a common complication of cytotoxic and vasogenic edema after ICH due to the inflammatory immune response and blood–brain barrier (BBB) destruction.^20^ A prospective cohort enrolling 176 ICH patients found a similar median PHE volume between prior statin users and non‐users before admission.^21^ However, this study was a retrospective analysis with a small sample size from a single‐center cohort. Enrollment and PHE measurements were also conducted at a period far from now (2009–2012), which may limit the generalizability of this study because of the changes in ICH medical management in recent years.

A pooled analysis combining the Intensive Blood Pressure Reduction in Acute Cerebral Hemorrhage Trial (INTERACT) studies indicated that absolute PHE growth was related to poor clinical outcomes in ICH.^22^ Considering that statins are beneficial in reducing PHE,^15^ they may be potential therapeutic agents for ICH. Moreover, different therapeutic strategies for statins may have different effects on ICH. A prospective cohort study enrolling 1275 patients with ICH found that continuation of prior statin therapy was safe, while initiating statin therapy during the first days after ICH may induce PHE growth.^23^ Continuous statin treatment was also associated with a lower risk of all‐cause mortality than discontinuous statin treatment.^24^

To elucidate the relationship between ICH and statins, investigation of their pathophysiological mechanisms is warranted, given that these mechanisms remain unclear. A meta‐analysis including 12 preclinical trials reported that statins were beneficial to significantly improve ICH recovery.^25^ A rat model of ICH also showed that statin use suppressed neutrophil mobilization and invasion, attenuating brain edema and neurological deficits after ICH.^26^ Statin use may also have neuroprotective antioxidant and BBB protective effects, including the acceleration of hematoma clean‐up post‐ICH by facilitating endogenous phagocytosis by erythrocytes.^26^ PHE includes vasogenic and cytotoxic edema caused by BBB damage and inflammation.^27^, ^28^ Considering that statins decreased the PHE volumes,^12^, ^15^, ^19^ statin use could reduce the severity and improve neurological recovery without increasing mortality in patients with ICH,^29^, ^30^, ^31^, ^32^, ^33^, ^34^ including in‐hospital mortality.^35^, ^36^

Several pathways have been identified to regulate neuroinflammation process and brain edema after ICH. Receptor‐interacting protein kinase‐1, a master regulator of neuroinflammation, mediates programmed necrosis (necroptosis) via the mixed‐lineage kinase‐like protein and induces brain edema after ICH.^37^ Irisin treatment has been shown to reduce brain edema through the integrin αVβ5/AMPK signaling pathway in mouse models.^38^ Lithium has also been reported to attenuate brain edema through the Wnt/β‐catenin signaling‐dependent mechanism in ICH mouse models.^39^ In addition, endogenously formed zinc protoporphyrin can induce ICH‐related damage, including the lysis of red blood cells and brain edema. Hence, ferrochelatase, a catalyst that inserts zinc into protoporphyrin, may be beneficial for reducing brain edema.^40^ Compared to the advances in therapeutic agents for ischemic stroke,^41^, ^42^, ^43^ progress in ICH research is limited and drugs targeted to brain edema, including statins, may be promising.

In patients without a history of dyslipidemia, prior statin use was associated with reduced in‐hospital mortality and intracranial hematoma evacuation rates. This relationship has not been established in patients with a history of dyslipidemia. In patients without dyslipidemia, the prescription of statins may be due to intra/extracranial or coronary artery stenosis caused by atherosclerotic plaques. A lack of a history of dyslipidemia indicated normal serum lipid levels and less damage to the blood vessel endothelium. With the addition of neuroprotection and anti‐inflammation of statins, the functional outcomes of ICH could be improved, manifesting as decreased mortality and a lower rate of evacuation of intracranial hematoma.^44^

Randomized clinical trials are warranted to clarify the impact of statins on ICH. The SATURN trial is a multicenter, pragmatic, prospective, randomized, open‐label, and blinded end‐point assessment (PROBE) clinical trial that aims to determine whether continuation or discontinuation of statin drugs after primary lobar ICH is the best strategy. The SATURN trial was designed to enroll 1456 participants and is estimated to be completed on December 31, 2026. The results of this study will help elucidate the relationship between statins and ICH using high‐quality evidence.

This study has several limitations. First, in contrast to a randomized controlled design, the retrospective design of the current study may have introduced selection bias. We performed logistic regression analyses and PSM to adjust for imbalanced covariates during statistical analysis. Second, the follow‐up information was lacking in the CSCA database. Assessments of long‐term outcomes, including functional independence, were unavailable. Third, the CSCA database was used to improve the quality of the diagnosis and treatment of cerebrovascular diseases in the neurology department. Some ICH patients may be admitted to the Department of Neurosurgery or the Intensive Care Unit. We may have a selection bias because patients included in the CSCA database may present with milder disease with a lower mortality rate. Fourth, we could not collect information on hematoma volume in our study, given that different methods are used to calculate hematoma volume in different sub‐centers of CSCA (manually, semiautomatic software, or automatic software). However, we compared the GCS scores between statin users and non‐users. Comparable GCS scores suggested that hematoma volume may be similar between the groups with prior statin use and without prior statin use. Fifth, we failed to collect information on the frequency and dose of oral statin agents, and future studies should include information on the frequency and dose.

In conclusion, prior statin use is associated with reduced rates of evacuation of intracranial hematoma but is not related to in‐hospital mortality in patients with ICH. Further randomized controlled trials are required to confirm these findings.

## AUTHOR CONTRIBUTIONS

X.Z., Z.L., and Y.X. contributed to the conception and design of the study; H.G., K.X., X.Y., C.W., and C.W. contributed to the acquisition and analysis of data; G.L. and S.W. contributed to drafting the text and preparing the figures.

## CONFLICT OF INTEREST

None.

## Data Availability

Data available on request from the authors.
